# Performance of node reporting and data system (node-RADS): a preliminary study in cervical cancer

**DOI:** 10.1186/s12880-024-01205-8

**Published:** 2024-01-26

**Authors:** Qingxia Wu, Jianghua Lou, Jinjin Liu, Linxiao Dong, Qingxia Wu, Yaping Wu, Xuan Yu, Meiyun Wang

**Affiliations:** 1 Department of Medical Imaging, Henan Provincial People’s Hospital, People’s Hospital of Zhengzhou University, People’s Hospital of Henan University, No. 7 Weiwu Road, Zhengzhou, Henan 450003 China; 2 Beijing United Imaging Research Institute of Intelligent Imaging, United Imaging Intelligence (Beijing) Co., Ltd., Beijing, 100089 China; 3 Laboratory of Brain Science and Brain-Like Intelligence Technology, Biomedical Research Institute, Henan Academy of Sciences, No. 266-38, Mingli Road, Zhengzhou, Henan 450046 China

**Keywords:** Cervical cancer, Lymph node, MRI, Metastasis

## Abstract

**Background:**

Node Reporting and Data System (Node-RADS) was proposed and can be applied to lymph nodes (LNs) across all anatomical sites. This study aimed to investigate the diagnostic performance of Node-RADS in cervical cancer patients.

**Methods:**

A total of 81 cervical cancer patients treated with radical hysterectomy and LN dissection were retrospectively enrolled. Node-RADS evaluations were performed by two radiologists on preoperative MRI scans for all patients, both at the LN level and patient level. Chi-square and Fisher’s exact tests were employed to evaluate the distribution differences in size and configuration between patients with and without LN metastasis (LNM) in various regions. The receiver operating characteristic (ROC) and the area under the curve (AUC) were used to explore the diagnostic performance of the Node-RADS score for LNM.

**Results:**

The rates of LNM in the para-aortic, common iliac, internal iliac, external iliac, and inguinal regions were 7.4%, 9.3%, 19.8%, 21.0%, and 2.5%, respectively. At the patient level, as the NODE-RADS score increased, the rate of LNM also increased, with rates of 26.1%, 29.2%, 42.9%, 80.0%, and 90.9% for Node-RADS scores 1, 2, 3, 4, and 5, respectively. At the patient level, the AUCs for Node-RADS scores > 1, >2, > 3, and > 4 were 0.632, 0.752, 0.763, and 0.726, respectively. Both at the patient level and LN level, a Node-RADS score > 3 could be considered the optimal cut-off value with the best AUC and accuracy.

**Conclusions:**

Node-RADS is effective in predicting LNM for scores 4 to 5. However, the proportions of LNM were more than 25% at the patient level for scores 1 and 2, which does not align with the expected very low and low probability of LNM for these scores.

**Supplementary Information:**

The online version contains supplementary material available at 10.1186/s12880-024-01205-8.

## Introduction

Lymph node metastasis (LNM) is one of the most significant prognostic factors in cervical cancer [1]. Patients with lymph node (LN) involvement are assigned as stage IIIC based on the FIGO 2018 system [2]. According to National Comprehensive Cancer Network (NCCN) guidelines, patients with FIGO IIIC are not candidates for radical hysterectomy and are recommended to undergo concurrent chemoradiation (CCRT) [3]. Additionally, patients with negative radiological LNs who undergo radical hysterectomy and LN dissection but are found to have positive pathological LNs still require adjuvant CCRT, which will result in increased complications and toxicities. Therefore, the accurate evaluation of LN status is crucial for treatment planning and prognosis prediction in patients with cervical cancer.

Until now, there have been many studies based on imaging characteristics for the precise evaluation of LN involvement [4–7]. However, the results have not been satisfactory [4]. Traditionally, size, morphology, and signal characteristics, as well as enhancement patterns, have been utilized to evaluate LNM [7]. Of which, size measurement, as a quantitative marker, is widely used in clinical applications, while the sensitivity is relatively low with metastasis in small LNs not being identified [6]. Functional MRI imaging, such as diffusion weighted imaging (DWI) and dynamic contrast enhanced (DCE) imaging, have shown added value in the diagnosis of LNM [5, 8]. DWI is helpful in detecting LNs, while the quantitative diffusion parameters in differentiating LNM are controversial [5, 6, 9], besides, the inconsistent b-value used among centers ranging from 600 to 2000 s/mm^2^ and various models developed for diffusion quantification limited the wide application of DWI quantitatively [5, 10–12]. DCE imaging allows not only rapid qualitative assessment but also quantitative measures of intrinsic perfusion and permeability parameters of target tissues [13]. Qualitative analysis of enhancement patterns is prone to errors due to the reader’s experience, the clinical use of semi-quantitative and quantitative DCE imaging remains limited outside of clinical trials [13]. Recently, radiomics analysis and machine learning, have emerged as promising tools for predicting LNM in cervical cancer [14, 15]. However, clinical translation still has a long way to go because of the explainability of the models, the reproducibility of the quantitative imaging features, and their sensitivity to variations in image acquisition and reconstruction parameters [16].

In 2021, Elsholtz FHJ et al. proposed a Node Reporting and Data System (Node-RADS) based on size and configuration, which includes texture, border, and shape of LNs [17]. This system is designed to be user-friendly and is guided by a three-tiered flowchart. The assessment of a LN using the Node-RADS scheme results in scores from 1 to 5. As the score increases, the probability of LNM also increases. Node-RADS has been validated in bladder cancer, lung cancer, and prostate cancer, demonstrating promising results [18–20]. To date, there have been no studies reporting the application and performance of this scoring system in cervical cancer. Since different tumors have specific lymphatic drainage pathways, and LN is the most common way for cervical cancer to spread [21], it is necessary to explore the evaluation of this scoring system in cervical cancer for future supplementation and improvement of the system. Therefore, this study retrospectively analyzed the performance of this scoring system in cervical cancer for LNM evaluation.

## Materials and methods

This retrospective study was approved by the ethics committee of the Hospital, and informed consent from patients was waived.

### Patients

**Fig. 1:**
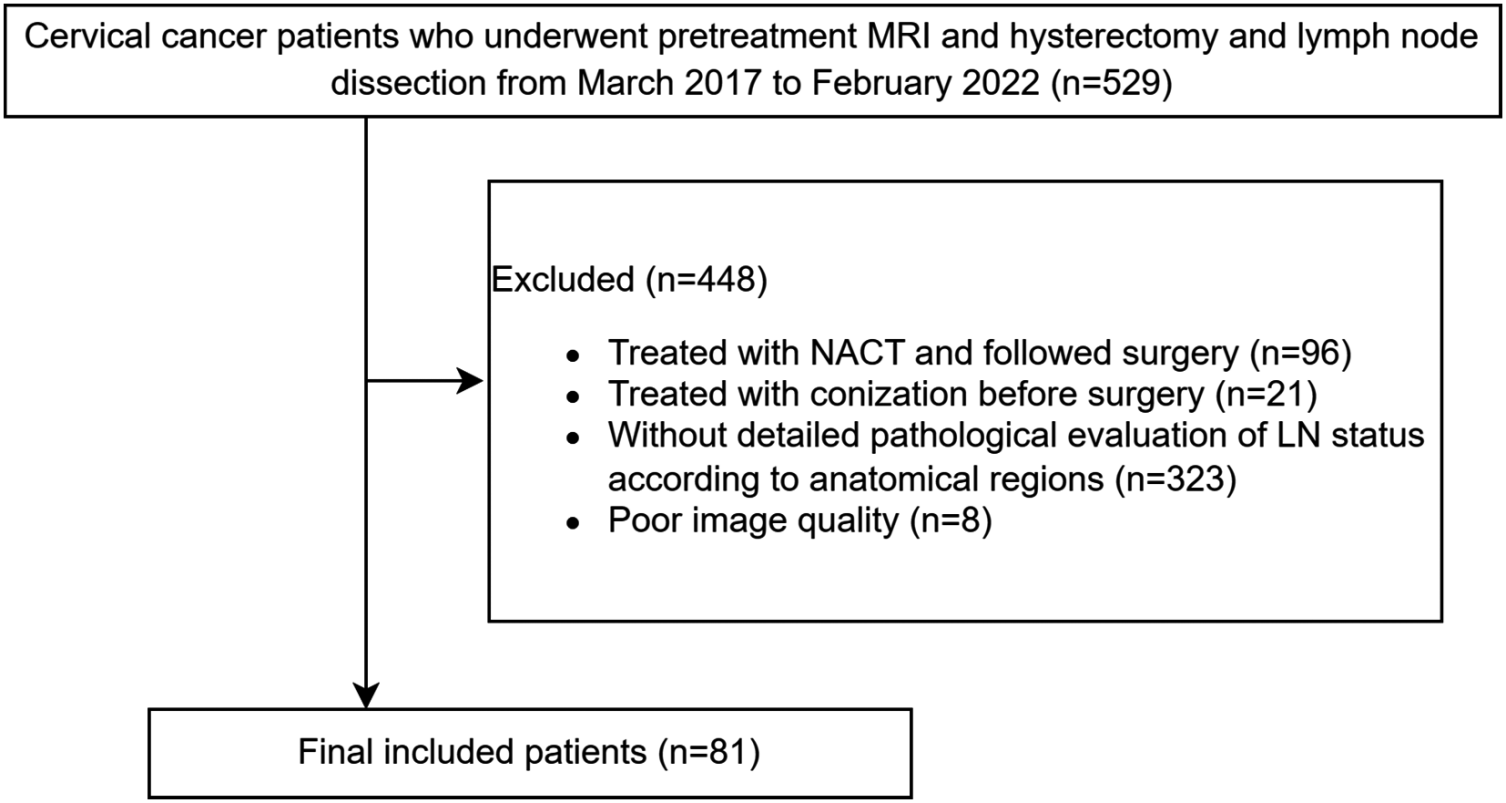
The patient recruitment flowchart

Cervical cancer patients who underwent radical hysterectomy and LN dissection treated at Henan Provincial People’s Hospital between March 2017 and February 2022 were screened for eligibility. The inclusion criteria consisted of (i) patients who underwent radical hysterectomy and LN dissection; (ii) patients who underwent pelvic MRI with gadolinium contrast for preoperative evaluation; (iii) the time interval between pelvic MRI and surgery was within 7 days; (iv) the pathological status of LNs were evaluated according to regions. The exclusion criteria were as follows: (i) patients underwent therapy including NACT or conization before surgery; (ii) the pathological evaluation of LN status was not detailed according to regions. The patient recruitment flowchart is shown in Fig. 1.

### MRI examination and node-RADS assessment

MRI scans were conducted using either one of the two scanners: Discovery MR 750 (GE Medical Systems, Milwaukee, WI, USA) or TrioTim (Siemens Healthcare). The imaging sequences and acquisition parameters can be found in the Supplementary Table.

Sagittal, coronal, and axial T2WI with or without fat saturation, axial T1WI, and axial DWI were acquired. Gadolinium was injected into the antecubital vein with 2mL/sec with a total amount of 0.2mL/kilogram. Then four T1WI with axial early-, late-arterial phases, and early-, late-venous phases with 18, 36, 54, and 72 s after gadolinium injection respectively were obtained. Finally, axial, coronal and sagittal T1WI 3 min after gadolinium injection as delayed phase were acquired.

Two radiologists (Jianghua Lou and Qingxia Wu, with 12 and 13 years of pelvic imaging experience respectively) evaluated the image findings separately on a dedicated Carestream software. Consensus was made if there was disagreement.

The readers were both blinded to the post-operative pathological results. The LN evaluation was performed according to Node-RADS recommendations. Typically, the LN with the largest short axis diameter was selected for evaluation in each region. However, if an LN in the region exhibited an abnormal texture, an irregular or ill-defined border, and a spherical shape without a fatty hilum, it was also evaluated. The LN with the highest score will then represent the LN in that region. The highest Node-RADS score among the nine regions will represent the Node-RADS score for the patient.

For size evaluation, LNs with a short axis diameter less than 10 mm were considered normal, those with a long axis diameter greater than 30 mm were considered bulk, and those falling between these two values were considered enlarged for para-aortic, bilateral common iliac, and internal iliac LNs. For bilateral external iliac LNs, if an obturator LN was evaluated, a short axis diameter less than 5 mm was considered normal, a short axis diameter greater than 5 mm and a long axis diameter less than 30 mm were considered enlarged, and a long axis diameter greater than 30 mm was considered bulk. For inguinal LNs, a short axis diameter less than 15 mm was considered normal, a short axis diameter greater than 15 mm and a long axis diameter less than 30 mm were considered enlarged, and a long axis diameter greater than 30 mm was considered bulk. For texture, border, and shape evaluation, Node-RADS recommendations are followed. Then, according to the three-level flowchart, the score for each evaluated LN in each region was calculated, resulting in an overall score ranging from 1 to 5. For the common iliac, internal iliac, external iliac, and inguinal LNs, both sides are evaluated and recorded. The LN with the highest Node-RADS score within a specific region is recorded as the score for the LNs within that particular region.

### Pelvic LN dissection and pathologic assessment

If bulky para-aortic nodes were identified on preoperative imaging or LN involvement was suspected during surgery, para-aortic lymphadenectomy was performed. Separate packets of LNs from different regions including bilateral inguinal, external, internal, and common iliac with or without para-aortic region were obtained. Specimens were evaluated according to our institution’s standard protocol. The tissue was fixed in 4% formalin and degreased in acetone. Suspicious nodes and tissue specimens were examined separately using palpation, visual inspection, and sectioning. All microscopically detected nodes were cut into 5 μm, embedded in paraffin, and stained with hematoxylin and eosin. The total number of histologically detected LNs and the number of positive nodes were reported in the pathological reports. Tumor histology type, differentiation, stromal invasion depth, lymphovascular space invasion (LVSI), perineural invasion, involvement of uterus corpus and vagina, and parametrial infiltration were retrieved from their pathological reports.

### Statistical analyses

Statistical analysis was performed with the SPSS software (version 22.0, IBM, Armonk, NY, USA) and GraphPad Prism (version 8.0.0, GraphPad Software, San Diego, CA, USA). The difference between patient groups with and without LNM was assessed using independent t test, Chi-square test, and Fisher’s exact test. Weighted Kappa test was used for the inter-observer agreement evaluation, the degree of agreement was classified as follows: perfect, κ ≥ 0.80; good, 0.60 ≤ κ < 0.80; moderate, 0.40 ≤ κ < 0.60; fair, 0.20 ≤ κ < 0.40; poor, κ < 0.20. The receiver operating characteristic (ROC) and the area under the curve (AUC) were used to explore the diagnostic performance of the Node-RADS score for LNM. The diagnostic ability was estimated for all possible cut-offs of the Node-RADS score (> 1 vs. >2 vs. >3 vs. >4).

## Results

### Patients’ characteristics

A total of 81 cervical cancer patients (mean age: 49 ± 10 years) underwent radical hysterectomy and LN dissection, as illustrated in Fig. 1. Among them, 29 (35.8%) underwent para-aortic LN biopsy or dissection. Clinical characteristics are detailed in Table 1. The median number of LNs removed per patient was 40.0 (IQR 29.5–51.5).

**Table 1:** Characteristics of cervical cancer patients with and without lymph node metastasis

Characteristics	All patients (*n* = 81)	LNM (−) (*n* = 41)	LNM (+) (*n* = 40)	P value
Age	49.8 ± 10.6	49.1 ± 10.1	50.4 ± 11.1	0.576
No. of LN removed	40.0(29.5–51.5)^§^	38.1 ± 14.9	41.0(32.3–52.5)^§^	0.184
Tumor size (cm)				1.000
≤ 2	5	3	2	
> 2,≤4	55	28	27	
> 4	21	10	11	
Tumor type				0.746
SCC	62	32	30	
NON-SCC	19	9	10	
Differentiation				0.829
Well and moderately	64	32	32	
Poorly	17	9	8	
Stromal invasion depth				0.244
≤ 1/3	8	5	3	
> 1/3, ≤ 2/3	34	20	14	
> 2/3	39	16	23	
LVSI				< 0.001
No	24	20	4	
Yes	57	21	36	
Perineural invasion				0.007
No	68	39	29	
Yes	13	2	11	
Corpus involvement				0.310
No	53	29	24	
Yes	28	12	16	
Vaginal involvement				0.312
No	72	38	34	
Yes	9	3	6	
Incision margin				0.012
No	75	41	34	
Yes	6	0	6	
Parametrial involvement				0.116
No	78	41	37	
Yes	3	0	3	

^§^Data are medians, with IQRs in parentheses

Since FIGO 2018 system allows pathological findings to allocate the stage, all the FIGO stages of patients were reevaluated to be consistent with FIGO 2018. After postoperative pathological evaluation, LNM was detected in 40 patients (49.4%), classifying them as IIICp. Within this group, there were 34 cases of IIICp1 and 6 cases of IIICp2. The radiological FIGO stages of the 40 IIICp patients were one IB1, eight IB2, one IB3, eight IIA1, five IIA2, 15 IIICr1 and two IIICr2, respectively. The correlation between pathological and radiological FIGO stages of these patients is illustrated in the Supplementary Figure. Among the 41 patients (50.6%) without LNM, there were seven IB1, 24 IB2, seven IB3, and three IIA1 patients, respectively.

Patients with LNM exhibited higher occurrences of lymphovascular space invasion (LVSI), perineural invasion, and incision margin involvement (all *p* < 0.05). Notably, the number of LNs removed showed no significant difference between patients with and without LNM (*p* = 0.184). The distribution of tumor size, tumor histology type, differentiation, stromal invasion depth, corpus, vaginal and parametrial involvement were balanced between patient groups with and without LNM. A total of 729 LNs distributed in the para-aortic, bilateral common iliac, internal iliac, external iliac, and inguinal regions were evaluated.

### Inter-observer agreement assessment

The weighted kappa value of Node-RADS score for LNs in para-aortic, common iliac, internal iliac, and external iliac regions were 0.829, 0.593, 0.547, and 0.556, respectively. In the inguinal region, the Node-RADS score was consistently rated as 1 for all patients, and therefore no value was obtained from the weighted kappa test. Regarding the assessment of the size, texture, border, and shape of all LNs, the weighted kappa values between the two readers were 0.677, 0.499, 0.425, and 0.363, respectively.

### Node-RADS score of LNs according to regions

The rates of LNM in different regions were as follows: 7.4% (6/81) in the para-aortic, 9.3% (15/162) in the common iliac, 19.8% (32/162) in the internal iliac, 21.0% (34/162) in the external iliac, and 2.5% (4/162) in the inguinal (Table 2). The size and configuration did not show discriminative ability for LNM in the inguinal regions. Size and shape are less reliable indicators of LNM compared to texture and border. The shape of LNs did not show a significant difference between patients with and without LNM in the para-aortic region, while the size of LNs did not show a significant difference between patients with and without LNM in the internal iliac region (Table 2).

**Table 2:** Comparison of characteristics between negative and positive lymph nodes in different regions

Characteristics	Para-aortic (*n* = 81)			Common iliac (*n* = 162)			Internal iliac (*n* = 162)			External iliac (*n* = 162)			Inguinal (*n* = 162)		
	LN (−)	LN (+)	P	LN (−)	LN (+)	P	LN (−)	LN (+)	P	LN (−)	LN (+)	P	LN (−)	LN (+)	P
**Size**			0.005			0.001			0.100			< 0.001			NA
Normal	75	4		147	12		129	30		69	13		158	4	
Enlarged	0	2		0	3		1	2		58	12		0	0	
Bulk	0	0		0	0		0	0		1	9		0	0	
**Configuration**															
**Texture**			0.013						< 0.001			< 0.001			1.000
Homogeneous	74	4		147	11	0.000	130	26		109	17		157	4	
Heterogeneous	1	1		0	0		0	1		11	3		0	0	
Focal necrosis	0	1		0	3		0	4		6	12		1	0	
Gross necrosis	0	0		0	1		0	1		2	2		0	0	
**Border**			0.005			0.006			< 0.001			0.009			1.000
Smooth	75	4		145	12		130	26		117	25		157	4	
Irregular or ill-defined	0	2		2	3		0	6		11	9		1	0	
**Shape**			0.144			0.001			< 0.001			< 0.001			1.000
Normal*	74	5		144	11		127	24		120	21		152	4	
Abnormal^	1	1		3	4		3	8		8	13		6	0	

*Any shape with preserved fatty hilum, or Kidney-bean-like or oval without fatty hilum

^Spherical shape without fatty hilum

**Fig. 2:**
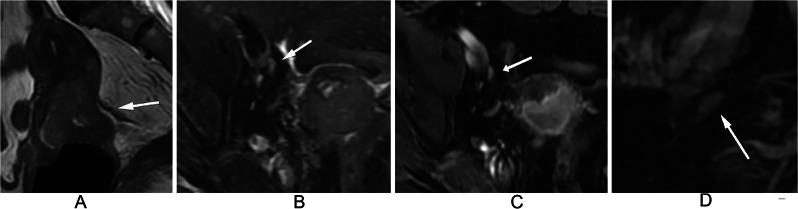
Sagittal T2WI (**A**) displays a 4.2 cm heterogeneous mass primarily situated in the posterior lip of the cervix. Axial T2WI with fat saturation (**B**), Axial T1WI with gadolinium (**C**), and Sagittal T1WI with gadolinium (**D**) reveal a normal-sized, smooth, homogeneous lymph node in the external iliac region, exhibiting an oval shape without a fatty hilum. Assigned a Node-RADS score of 1, this lymph node, the largest in this area, represents the Node-RADS score for the external iliac region. Pathological evaluation confirmed it as a negative lymph node

**Fig. 3:**
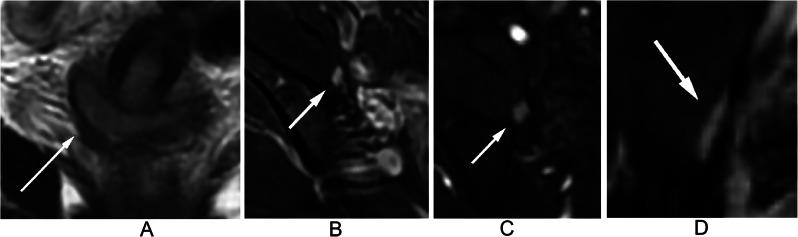
Coronal T2WI (**A**) displays a 3.0 cm mass primarily situated in the right cervix. Axial T2WI with fat saturation (**B**), Axial T1WI with gadolinium (**C**), and Coronal T1WI with gadolinium (**D**) reveal a normal-sized, smooth, homogeneous lymph node in the external iliac region, exhibiting an oval shape without a fatty hilum. Assigned a Node-RADS score of 1, this lymph node, the largest in this area, represents the Node-RADS score for the external iliac region. Pathological evaluation confirmed it as a positive lymph node

**Fig. 4:**
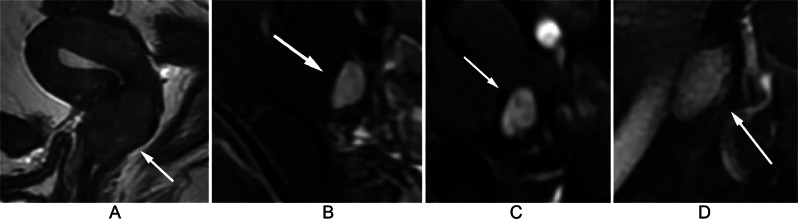
Sagittal T2WI (**A**) displays a 3.6 cm mass in the cervix. Axial T2WI with fat saturation (**B**), Axial T1WI with gadolinium (**C**), and Sagittal T1WI with gadolinium (**D**) reveal an enlarged, heterogeneous lymph node in the external iliac region, with a smooth border, a short axis diameter of 10 mm, and an oval shape without a fatty hilum. Assigned a Node-RADS score of 3, this lymph node, the largest in this area, was chosen to represent the Node-RADS score for the external iliac region. Pathological evaluation confirmed it as a negative lymph node

**Fig. 5:**
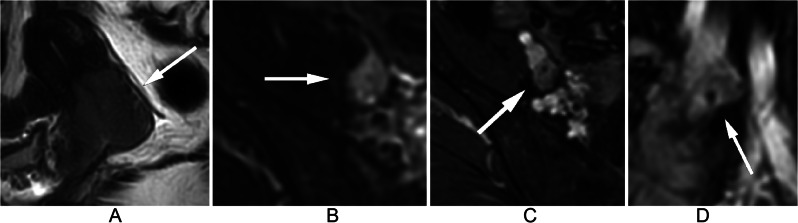
Sagittal T2WI (**A**) displays a 5.4 cm mass in the cervix. Axial T2WI with fat saturation (**B**), Axial T1WI with gadolinium (**C**), and Sagittal T1WI with gadolinium (**D**) reveal an enlarged lymph node in the external iliac region, with a short axis diameter of 12 mm. There is focal necrosis inside the lymph node and the border is a little irregular (**C**). The lymph node appears triangular without a fatty hilum. Assigned a Node-RADS score of 5, this lymph node, the largest in this area, was selected to represent the Node-RADS score for the external iliac region. Pathological evaluation confirmed it as a positive lymph node

At the node level, 5.1%, 7.1%, 15.9%, 15.0%, and 2.6% of LNs with a NODE-RADS score of 1 were found to be metastatic in the para-aortic, iliac, internal iliac, external iliac, and inguinal regions, respectively. Among LNs with a NODE-RADS score of 2 in the internal iliac and external iliac regions, metastatic was found to be 40.0% and 9.3% respectively. Figure 2 shows a negative LN with a Node-RADS score of 1 located in the external iliac region, while Fig. 3 displays a positive LN with the same Node-RADS score located in the same region. Surprisingly, none of the LNs with a NODE-RADS score of 3 were found to be metastatic. LNs with NODE-RADS scores of 4 and 5 tended to be metastatic, with 65.4% of LNs with a NODE-RADS score of 5 in the external iliac region being metastatic. Figure 4 shows a negative LN with a NODE-RADS score of 3, whereas Fig. 5 presents a positive LN with a NODE-RADS score of 5, both located in the external iliac region (Table 3).

**Table 3:** Node-RADS diagnostic information at lymph node level in different regions

NODE-RADS	Para-aortic (*n* = 81)			Common iliac (*n* = 162)			Internal iliac (*n* = 162)			External iliac (*n* = 162)			Inguinal (*n* = 162)		
	LN (−)	LN (+)	PR (%)	LN (−)	LN (+)	PR (%)	LN (−)	LN (+)	PR (%)	LN (−)	LN (+)	PR (%)	LN (−)	LN (+)	PR (%)
1	74	4	5.1	143	11	7.1	127	24	15.9	68	12	15.0	151	4	2.6
2	0	0	0.0	3	0	0.0	3	2	40.0	39	4	9.3	7	0	0.0
3	1	0	0.0	1	0	0.0	0	0	0.0	8	0	0.0	0	0	0.0
4	0	0	0.0	0	1	100.0	0	6	100.0	4	1	20.0	0	0	0.0
5	0	2	100.0	0	3	100.0	0	0	0.0	9	17	65.4	0	0	0.0

At the patient level, an increase in the NODE-RADS score corresponded to an increased rate of LNM. The rates were as follows: 26.1% for Node-RADS score 1, 29.2% for Node-RADS score 2, 42.9% for Node-RADS score 3, 80.0% for Node-RADS score 4, and 90.9% for Node-RADS score 5. For patients with squamous cell carcinoma (SCC), the rates of LNM were 26.3%, 38.9%, 20.0%, 75.0%, and 87.5% for Node-RADS scores 1, 2, 3, 4, and 5, respectively. For patients with non-SCC, the rate of LNM for Node-RADS score 1 was found to be at a constant value of 25.0%. However, the rates reached 100.0% for patients with Node-RADS scores of either 3, 4, or 5 respectively. None of the patients in the non-SCC group with a Node-RADS score 2 showed LNM (Table 4).

**Table 4:** Node-RADS diagnostic information at patient level

NODE-RADS	All patients (*n* = 81)			SCC patients (*n* = 62)			Non-SCC patients (*n* = 19)		
	LN (−)	LN (+)	PR (%)	LN (−)	LN (+)	PR (%)	LN (−)	LN (+)	PR (%)
1	17	6	26.1	14	5	26.3	3	1	25.0
2	17	7	29.2	11	7	38.9	6	0	0.0
3	4	3	42.9	4	1	20.0	0	2	100.0
4	1	4	80.0	1	3	75.0	0	1	100.0
5	2	20	90.9	2	14	87.5	0	6	100.0

### Diagnostic performance of the node-RADS score

Sensitivity, specificity, positive predictive value (PPV), and negative predictive value (NPV) for LNM when different Node-RADS scores were used are shown in Table 5.

**Table 5:** Performance of different Node-RADS cut-offs for the diagnosis of LNM at patient and LN level

Node-RADS cut-off	AUC	Sensitivity (%)	Specificity (%)	PPV (%)	NPV (%)	Accuracy (%)
**Patient level**						
> 1	0.632	85.0	41.5	58.6	73.9	62.9
> 2	0.752	67.5	82.9	79.4	72.3	75.3
> 3	0.763	60.0	92.7	88.9	70.4	76.5
> 4	0.726	50.0	95.1	90.9	66.1	72.8
**LN level**						
> 1	0.639	39.6	88.2	32.4	91.1	82.2
> 2	0.647	33.0	96.4	56.6	91.0	88.5
> 3	0.655	33.0	98.0	69.8	91.1	89.8
> 4	0.614	24.2	98.6	71.0	90.1	89.3

**Fig. 6:**
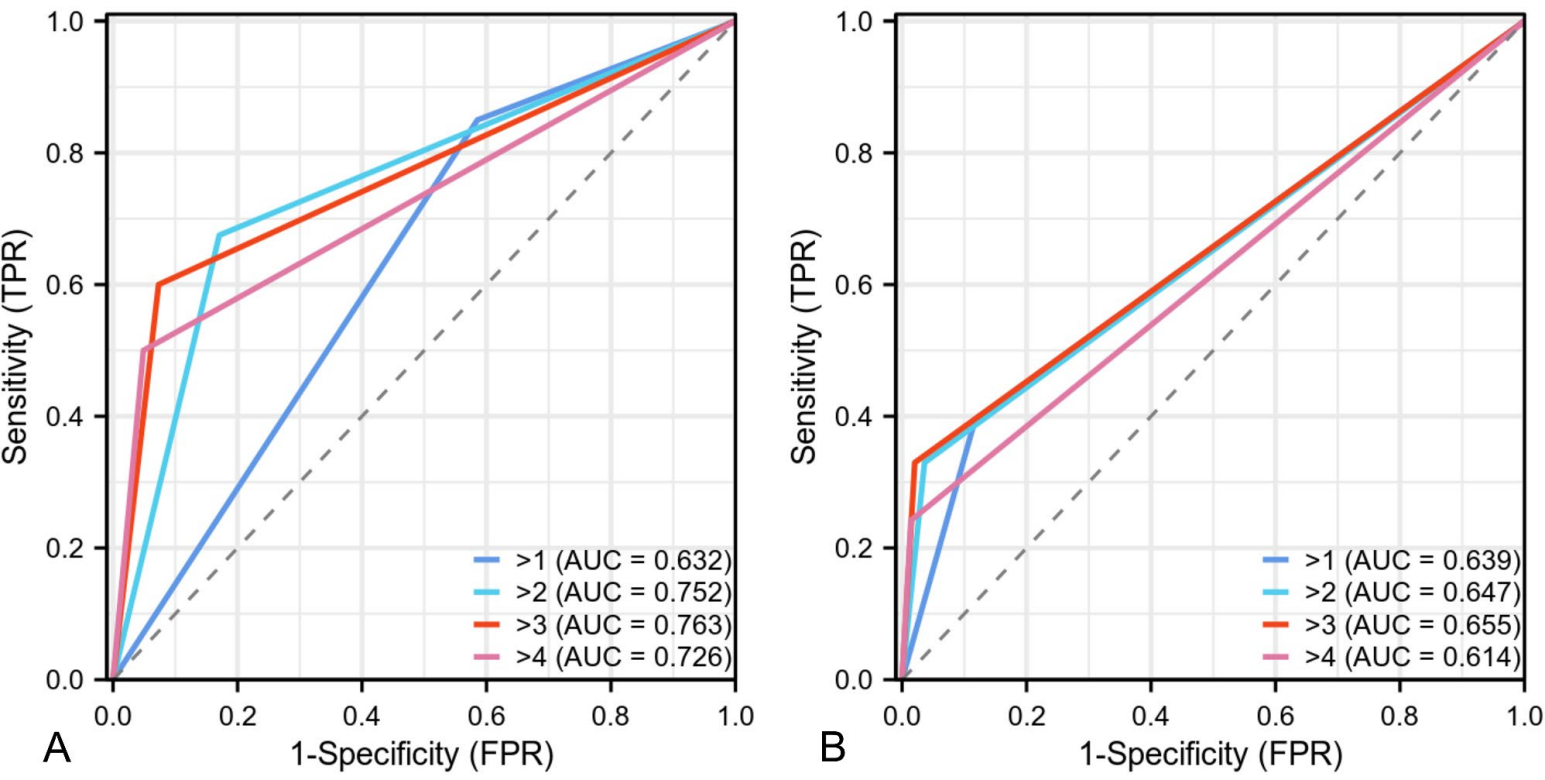
Receiver operating characteristic (ROC) curves at different Node-RADS score cut-offs for lymph node metastasis (LNM) at patient level (**A**) and lymph node level (**B**)

At the patient level, the AUCs for Node-RADS scores > 1, >2, > 3, and > 4 were 0.632, 0.752, 0.763, and 0.726, respectively (Fig. 6). As the cut-off value increased, the sensitivity decreased from 85.0 to 50.0%, while the specificity increased from 41.5 to 95.1% (Table 5). Similar trends were observed at the LN level. However, at the LN level, the sensitivity for LNM was extremely low for all Node-RADS cut-off values, with the highest sensitivity of 39.6% observed when Node-RADS > 1 and the lowest sensitivity of 24.2% observed when Node-RADS > 2. Both at the patient level and LN level, a Node-RADS score > 3 could be considered the optimal cut-off value due to its superior AUC and accuracy levels.

## Discussion

In our study, we applied Node-RADS for the first time to evaluate LNM in cervical cancer and reported the probability of LNM for different Node-RADS scores. Our results showed that Node-RADS is effective in predicting LNM for scores ranging from 4 to 5. However, we observed that the proportions of LNM exceeded 25% at the patient level for scores 1 and 2, which does not align with the expected very low and low probability of LNM for these scores.

Size, especially the short diameter of LNs, is widely used in evaluating LNs, with the highest accuracy compared with shape, texture, and border [7, 22]. The utilization of size is also favored due to its quantitative nature, ensuring a high inter-observer agreement. Whereas qualitative parameters such as texture, border and shape exhibit only fair and moderate inter-observer agreement both in our study and previous studies [4, 7, 19]. As for the shape of LNs, the quantitative short-to-long axis ratio was widely used, and it was proved to be less effective than size [6, 22]. The inhomogeneous signal intensity or central necrosis had a high PPV in the diagnosis of LNM [7, 22]. Combining size and morphology resulted in better diagnostic ability for LNM in pancreatic ductal adenocarcinoma [23]. Notably, Node-RADS is such a structured reporting system incorporating size, shape, texture, and border for the first time. It classifies LNs into scores ranging from 1 to 5 and can be applied across all anatomical sites. In the cervical cancer of our study, the proportions of LNM were 26.1% in patients with score 1 and 29.2% in patients with score 2, which does not align with the expected very low and low probability of LNM for these scores. This disparity arises since Node-RADS is primarily based on size criteria, while it is worth noting that 30-50% of metastatic LNs in pelvic cancers are less than 10 mm in the short axis [10]. Based on a previous study, about 63.4% of LNM in cervical cancer were smaller than 10 mm [6]. About 77.3%, 68 LNs with less than 3 mm in short-axis diameter out of 88 normal-sized LNs with a short-axis diameter of less than 8 mm proved to be metastatic in patients with bladder or prostate cancers [5]. Normal-sized LNs usually appear homogeneous and possess smooth borders and fatty hilum or oval shape and this will result in a Node-RADS score of 1, and even with spherical shapes resulting in a Node-RADS score of 2, some of them may still be metastatic. This elucidates the insufficiency in the predictive capacity for LNM associated with Node-RADS scores of 1 and 2, suggesting efforts should be made to improve the performance of the Node-RADS system.

Node-RADS sets different criteria for LNs depending on the regions. Generally, LNs with short axis larger than 10 mm were considered enlarged. For obturator LNs, a short axis of more than 5 mm is considered enlarged, while in the inguinal region, an LN with a short axis greater than 15 mm is considered enlarged. However, even with the specific relatively low criteria for obturator LNs, which serve as the sentinel LN in cervical cancer, Node-RADS scores of 1 and 2 did not perform well in evaluating LNM.

A comparison of LN features in different regions reveals that the size and configuration did not work well with inguinal LNs. Only approximately 2.5% of LNM at LN level occurred in the inguinal regions, by employing a criteria of a short axis of 15 mm for enlargement, none of the LN in the inguinal region was enlarged. And metastatic LN in the inguinal regions did not show abnormal texture, border and shape. This suggests the need for improvements in evaluating inguinal LNs in cervical cancer.

Leonardo C et al. recruited 49 bladder cancer patients to evaluate the performance of Node-RADS for LNM and found that 0%, 30.0%, 50.0%, 42.9%, and 83.3% of patients with scores 1, 2, 3, 4, and 5 showed LNM respectively [18]. Node-RADS > 2 was considered the optimal cut-off value due to balanced performance both in bladder cancer and lung cancer [18, 19]. However, Lucciola S found that the sensitivity of Node-RADS score > 3 for LNM prediction is 22.2% in prostate cancer [20], which limits the clinical implication of Node-RADS for pretreatment evaluation of LN status.

Radiomics analysis or machine learning based on primary tumors or LNs showed promising results for LNM prediction, especially increasing the sensitivity for LNM [10, 14]. Zhang Yu et al. found that traditional MRI features of LN such as short axis, long axis, short to long axis ratio, margin, and enhancement pattern did not help in the prediction of LNM, while both the radiomics features of LNs and primary tumor showed potential in the prediction of LNM [10]. It is worth noting that in small LNs, the extraction of radiomics features may be limited due to insufficient pixel data, and there is also uncertainty regarding whether the chosen LNs for radiomics feature extraction precisely correspond to pathologically positive LNs. Consequently, we believe that radiomics analysis of primary tumors will be a direction for LNM prediction in the future.

There are several limitations in this study. First, the retrospective nature and the lack of a specific imaging-pathological correlation do not allow a reliable per-lesion analysis. Second, the size of each LN was not specified in the pathological reports. The resected LNs far outnumber the LNs evaluated on MRI in our study. Usually, we choose LNs with the largest size or abnormal border, texture, and shape for the highest Node-RADS score to represent the LNs in that region. As a retrospective study, we cannot guarantee the LNs evaluated on MRI are exactly the pathologically positive LNs. Last, considering the relatively small number of patients in this single center, we did not evaluate the added value of radiomics analysis of primary tumors for LNM prediction.

## Conclusion

Node-RADS is effective in predicting LNM for scores ranging from 4 to 5. However, the proportions of LNM exceeding 25% at the patient level were observed for scores 1 and 2, which does not align with the expected very low and low probability of LNM for these scores.

### Electronic supplementary material

Below is the link to the electronic supplementary material.

Supplementary Material 1

## Data Availability

The datasets used and/or analysed during the current study are available from the corresponding author on reasonable request.

## Abbreviations

LNM: Lymph node metastasis
LN: Lymph node
NCCN: National Comprehensive Cancer Network
CCRT: Concurrent chemotherapy and radiotherapy
DWI: Diffusion weighted imaging
DCE: Dynamic contrast enhanced
Node-RADS: Node Reporting and Data System
ROC: Receiver operating characteristic
AUC: Area under ROC curve
LVSI: Lymphovascular space invasion
SCC: Squamous cell carcinoma
PPV: Positive predictive value
NPV: Negative predictive value
