# Supplementary material for: Performance of node reporting and data system (node-RADS): a preliminary study in cervical cancer
Source: BMC Med Imaging. 2024 Jan 26;24:28. doi: 10.1186/s12880-024-01205-8 (PMC10811875; doi:10.1186/s12880-024-01205-8)

**Supplementary table**

MRI protocols

| **Scanner** | **Sequence** | **TR (ms)** | **TE (ms)** | **Slice Thickness (mm)** | **Slice Gap (mm)** | **Bandwidth** | **Slices** |
| --- | --- | --- | --- | --- | --- | --- | --- |
| GE 3.0T  (Discovery MR 750) | Sagittal T2 | 4000 | 87 | 5 | 1 | 163 | 20 |
|  | Coronal T2 | 3927 | 81 | 5 | 1 | 122 | 20 |
|  | Axial T2 | 4167 | 87 | 5 | 2 | 139 | 22 |
|  | Axial T1 | 446 | 7 | 5 | 2 | 325 | 22 |
|  | Axial DWI | 2600 | 74 | 5 | 2 | 1953 | 22 |
|  | Axial T1 C+ | 4 | 2 | 2 | 0 | 558 | 108 |
|  | Sagittal T1C+ | 4 | 2 | 1.5 | 0 | 558 | 96 |
|  | Coronal T1C+ | 4 | 2 | 2 | 0 | 651 | 64 |
|  |  |  |  |  |  |  |  |
| SIEMENS 3.0T  (TrioTim) | Sagittal T2 | 3000 | 96 | 4 | 1.2 | 250 | 21 |
|  | Coronal T2 | 4000 | 104 | 5 | 1 | 203 | 20 |
|  | Axial T2 | 4000 | 87 | 5 | 1.5 | 250 | 21 |
|  | Axial T1 | 700 | 11 | 5 | 1.5 | 203 | 21 |
|  | Axial DWI | 4500 | 76 | 5 | 1.5 | 1736 | 21 |
|  | Axial T1 C+ | 3.3 | 1.1 | 1.5 | 0 | 501 | 96 |
|  | Sagittal T1C+ | 3.3 | 1.2 | 2 | 0 | 501 | 96 |
|  | Coronal T1C+ | 3.3 | 1.1 | 1.5 | 0 | 501 | 96 |
|  | SagittalT1C+ | 6.3 | 3.0 | 2.5 | 0 | 520 | 44 |

**Supplementary Figure** The correlation between pathological and radiological FIGO stages of IIICp patients. The number following the horizontal line in each stage indicates the count of patients with the respective FIGO stage.


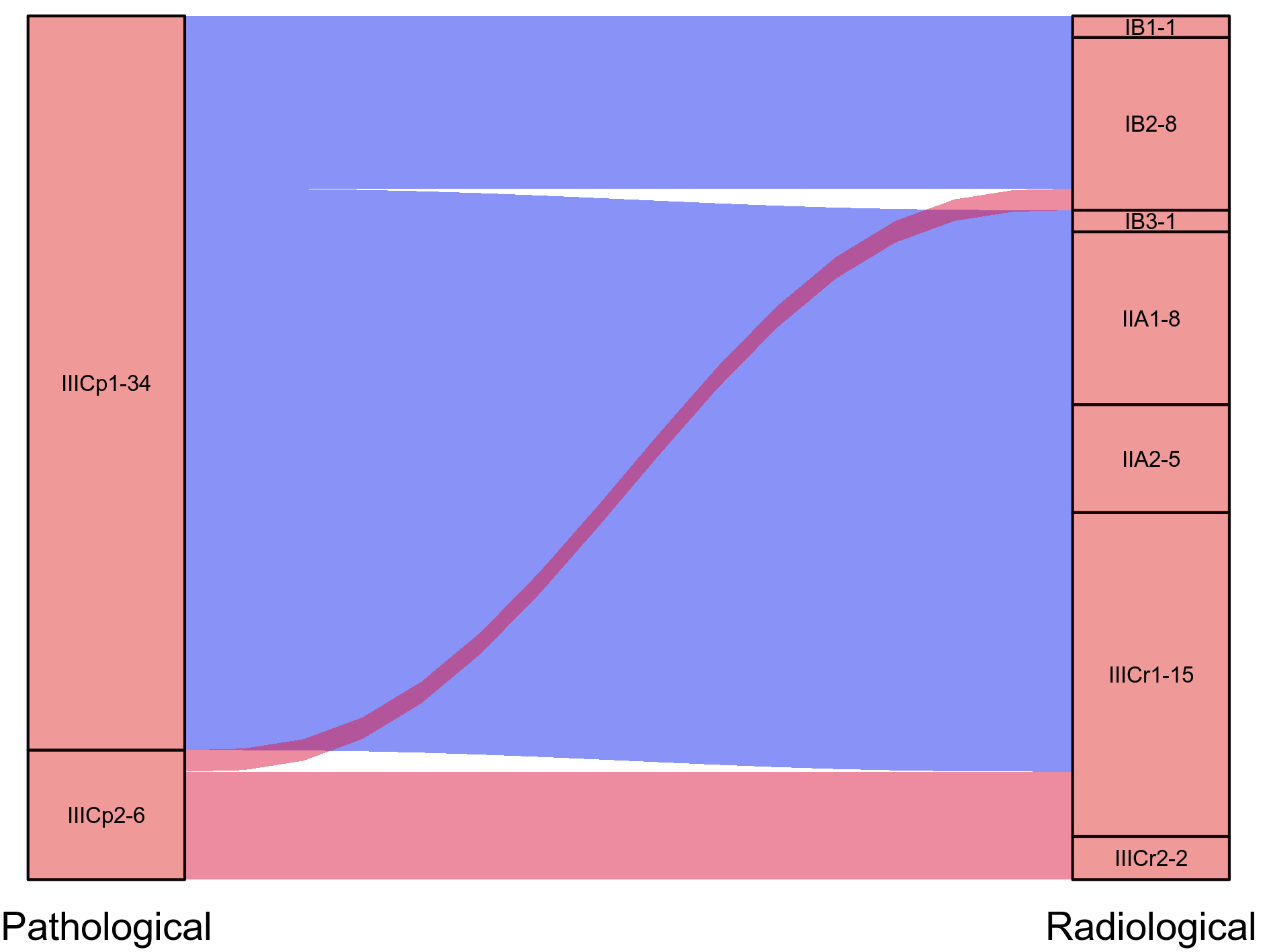

Supplement: Supplementary file 1 — Supplementary Material 1 [file 12880_2024_1205_MOESM1_ESM.docx]
